# *Xylopia aethiopica* ethanol seed extract suppresses Cadmium chloride-induced ovary and gonadotropins toxicity in adult female Wistar rats

**DOI:** 10.5935/1518-0557.20200091

**Published:** 2021

**Authors:** Elvis T Godam, Olugbemi T Olaniyan, Chiwendu D Wofuru, Clinton D Orupabo, Kenneth S Ordu, Barinua K Gbaranor, Progress D Dakoru

**Affiliations:** 1 Department of Human Anatomy, Faculty of Basic Medical Sciences, College of Medical Sciences, Rivers State University, Port Harcourt, Nigeria; 2 Laboratory for Reproductive Biology and Developmental Programming, Department of Physiology, Edo University Iyamho, Nigeria; 3 Department of Medical Laboratory Sciences, Faculty of Sciences, Rivers State University, Port Harcourt, Nigeria; 4 Department of Human Physiology, Faculty of Basic Medical Sciences, College of Medical Sciences, Rivers State University, Port Harcourt, Nigeria

**Keywords:** *Xylopia aethiopica*, cadmium chloride, toxicity, gonadotropins, Wistar rats

## Abstract

**Objective:**

*Xylopia aethiopica* is a common plant in West Africa, with wide applications in trado-medical management of several diseases. Thus, our study aimed to analyze the histology and hormonal effects of ethanol extracts of *Xylopia aethiopica* seeds on cadmium chloride-induced reproductive dysfunction in female Wistar rats.

**Methods:**

We used twenty-five rats weighing 120-150g for this study. The rats were divided into five groups (n=5). Group 1: received only distilled water orally; Group 2: received 2 mg/kg cadmium chloride orally; Group 3: received 2 mg/kg cadmium chloride plus 50 mg/kg *Xylopia aethiopica* seeds orally; Group 4: received 2 mg/kg cadmium chloride plus 100 mg/kg *Xylopia aethiopica* seeds orally, and Group 5: received 100 mg/kg *Xylopia aethiopica* seeds only, orally. We administered the extracts for 14 days, after which we slaughtered the animals following chloroform anesthesia. We took the blood samples by cardiac puncture for hormonal assay. The ovaries and uterus were harvested for histology. We analyzed the data using ANOVA, and the differences in mean values were considered significant at *p*<0.05.

**Results:**

The body weight of the rats showed a dose-dependent reduction (*p*<0.05), compared with the controls. *Xylopia aethiopica* seeds significantly (*p*<0.05) reversed the detrimental effects of Cadmium on LH and FSH. The histological analysis of the ovary showed significant improvement upon treatment with *Xylopia aethiopica* extract in a dose-dependent manner.

**Conclusions:**

The ameliorative effects of *Xylopia aethiopica* against cadmium chloride-induced reproductive toxicity in female Wistar rats may be attributed to its antioxidant properties.

## INTRODUCTION

Pharmaceutical companies in the production of vaccines, drugs and other health supplements utilize plants and their derivatives. About 30% of modern drugs are derived from plant-based metabolites or biomolecules (Burns, 2000). Studies have shown that about 80% of the African population utilize traditional or medicinal plants as a major means of healthcare needs (Johnson *et al.*, 2007). According to the World Health Organization, it has been estimated that about 75% of the world’s population use herbal remedies, especially owing to the fact that it possess fewer side effects compared to orthodox drugs (Liu *et al.*, 2007; Desai *et al.*, 2009).

One of the several West and Central Africa plants with great therapeutic properties is *Xylopia aethiopica,* widely utilized by traditional medicine practitioners in the management of numerous diseases. *Xylopia aethiopica* is a slim tree, about 60-70 cm in diameter, 15-30 m tall, mainly found growing in the tropical forests of Ghana, Nigeria and Cameroon. The seeds outlines are noticeable from outside, and each pod holds about 5 to 8 kidney-shaped seeds with approximately 5 mm in length (John Kennedy *et al.*, 2011). In Nigeria, it is commonly used as spice in the preparation of two special local soups named “Obe ata” and “Isi-ewu”, grown widely in the Southern parts of Nigeria (Tairu *et al*., 2000). Traditionally, it has been utilized for inducing placental discharge postpartum, management of rheumatism, asthma, headache, bronchitis, neuralgia and colic pain (Woode *et al*., 2011). In addition, the fruit is applied in combination with other herbal remedies, like *Piper nigrum*, *Capsicum frutescence* plus *Tetrepleura tetraptera* for newly delivered mothers; it helps in uterine contraction (Durugbo, 2013). Studies have revealed that the fruit has anti-malaria, antibacterial and antifungal properties.

Cadmium chloride is an endocrine disruptor, with detrimental effects to the female reproductive system, and human exposure is usually through inhalation plus ingestion of contaminated products (Ansa *et al*., 2017). Cadmium chloride is discharged into the environment from different sources of industrial activities causing health hazard, and posing as environmental risk to both humans and animals (Orisakwe, 2014). Cadmium chloride levels in humans can be confirmed via blood, saliva, urine and organ analyses. The blood analysis detects recent exposures to cadmium while urine analysis detects accurately both recent and past exposures to cadmium (Jin *et al*., 2002). Cadmium chloride toxicity can affects multiple organs such as the kidneys, muscles, liver, reproductive system, lungs and the cardiovascular system (Dix-Cooper & Kosatsky, 2018). Studies have revealed that cadmium chloride inhibits progesterone and estrogen production by blocking the gene expression of the steroidogenic acute regulatory protein StAR, plus cytochrome P450 cholesterol side chain enzyme P450scc (Pollack *et al*., 2014). This protein facilitates intra mitochondrial movement of cholesterol (Manna *et al*., 2016). Therefore, the aim of the study is to investigate the histology and hormonal effects of ethanol seed extracts of *Xylopia aethiopica* on cadmium chloride-induced reproductive toxicity in female Wistar rats.

## MATERIALS AND METHODS

We had twenty-five female Wistar rats weighing between 120-150g, from the Animal Housing facility, Department of Pharmacology, College of Medicine and Health Sciences, University of Port Harcourt, Nigeria. They were acclimatized for two weeks before starting the experiment, in accordance with the standard guide for the care and use of laboratory animals.

### Plant collection and Preparation

Samples of the leaves and seeds were identified at the Herbarium of the Department of Biological Sciences, Ahmadu Bello University, Zaria, Nigeria, where the voucher specimen number 547 was deposited. The air-dried seeds of *Xylopia aethiopica* were extracted by cold maceration in ethanol, and evaporated to dryness on a rotary evaporator (rotavap R-200), at a reduced temperature.

### Experimental protocol

The animals were randomly divided into five groups of five rats each, as follows:

Group 1- control animals received only distilled water orally.

Group 2- Animals given oral doses of 2 mg/kg body weight of cadmium chloride.

Group 3- Animals treated with 2 mg/kg body weight of cadmium chloride plus 50mg/kg body weight of *Xylopia aethiopica* extract orally.

Group 4- Animals treated with 2 mg/kg body weight of cadmium chloride plus 100mg/kg body weight of *Xylopia aethiopica* extract orally.

Group 5- Animals treated with 100 mg/kg body weight of *Xylopia aethiopica* extract only.

### Sample Collection

We administered the extracts for 14 days after which, the animals were sacrificed following chloroform anesthesia. Blood samples were taken by cardiac puncture from all groups for hormonal assay (Follicle Stimulating Hormone and Luteinizing Hormone). The ovaries and uteri were harvested after abdominal incision, and then fixed in 10% buffered formalin. Tissue sections were prepared using routine histological tissue preparation. Photomicrographs were taken using the 5 mega pixel Amscope digital scope, mounted on an Olympus microscope.

### Estimation of serum luteinizing hormone, and Follicle Stimulating Hormone

The serum samples obtained were analyzed to determine the concentrations of luteinizing hormone and follicle stimulating hormone. The analysis was carried out via the tube-based enzyme immunoassay (EIA) method. The protocol used in hormone testing followed the method described by the kit manufacturers (Immunometrics Limited UK), and met the WHO research program standards for reproductive studies.

### Histological Analyses

Histological investigations were carried out on the tissues fixed in 10% buffered formalin. The tissue blocks were sectioned for routine Hematoxylin and Eosin (H&E) staining. The fixed organs were cut in about 0.5cm cross-sections and transferred to 70% alcohol for dehydration. The tissues were rinsed in 90% and absolute alcohol and xylene for different durations before they were transferred into two changes of molten paraffin wax for 1 hour each, in an oven at 65ºC for infiltration. They were subsequently embedded and sliced in serial sections using a rotary microtome at five microns (5µ). The tissues were transferred onto albumenized slides and allowed to dry on a hot plate for 2 minutes. The slides were dewaxed with xylene and passed through absolute alcohol (2 changes); 70% alcohol, 50% alcohol, and then water for 5 minutes. The slides were then stained with hematoxylin and eosin.

### Statistical Analysis

The data was presented as mean values ± SEM. ANOVA was carried out on the Statistical Package for the Social Sciences (SPSS package version 17) and we checked for the occurrence of significant differences between the results. Differences were considered significant at *p*<0.05.

## RESULTS

### Effects *Xylopia aethiopica* treatment on the body weight in Cadmium chloride-induced Reproductive toxicity in Wistar rats

The results obtained showed that *Xylopia aethiopica* treatment caused significant increase (*p*<0.05) in the body weight compared with the control and cadmium chloride treated group (Table 1).

**Table 1:** Effect of treatment with seed extracts of *Xylopia aethiopica* on body weight (g) in Cadmium chloride-induced Reproductive toxicity in Wistar rats.

Group	Week 1	Week 2	Week 3	Week4
Group 1	83.40±2.66	101.80±2.52	139.00±1.87	143.40±2.14
Group 2	89.00±2.80	106.00±1.87	120.00±2.55^a^	100.00±1.87^a^
Group 3	86.40±3.23^b^	99.20±2.96^b^	114.00±1.87^b^	99.20±1.66^b^
Group 4	86.00±6.00	104.60±5.58	122.00±3.39^b^	111.80±2.46^b^
Group 5	87.00±5.39	104.40±3.06	129.400±1.69	120.00±4.11

Values are expressed as mean ± SEM, n=5. ^a,b^denote significant variation (*p*<0.05) when compared with control and cadmium chloride groups.

### Effects *Xylopia aethiopica* treatment on the hormonal activity in Cadmium chloride-induced Reproductive toxicity in Wistar rats

The results obtained in Table 2 showed that the serum LH and FSH levels were significantly increased (*p*<0.05) by treatment with Cadmium chloride while *Xylopia aethiopica* seeds extract showed a significant reduction in (*p*<0.05) the level of LH and FSH.

**Table 2:** Effect of treatment with seeds extracts of *Xylopia aethiopica* on hormonal activity in Cadmium chloride-induced Reproductive toxicity in Wistar rats

Group	FSH(mIU/mL)	LH (mIU/mL)
Group 1	0.24±0.02	0.26±0.03
Group 2	6.25±0.70^a^	6.11±0.75^a^
Group 3	3.41±0.03^b^	3.30±0.04^b^
Group 4	0.16±0.02^b^	0.17±0.02^b^
Group 5	0.15±0.03^a^	0.15±0.03^a^

Values are expressed as mean ± SEM, n=5. ^a,b^denote significant variation (p<0.05) when compared with control and cadmium chloride groups.

### Effects *Xylopia aethiopica* treatment on the histology in Cadmium chloride-induced Reproductive toxicity in Wistar rats

**Figure 1:**
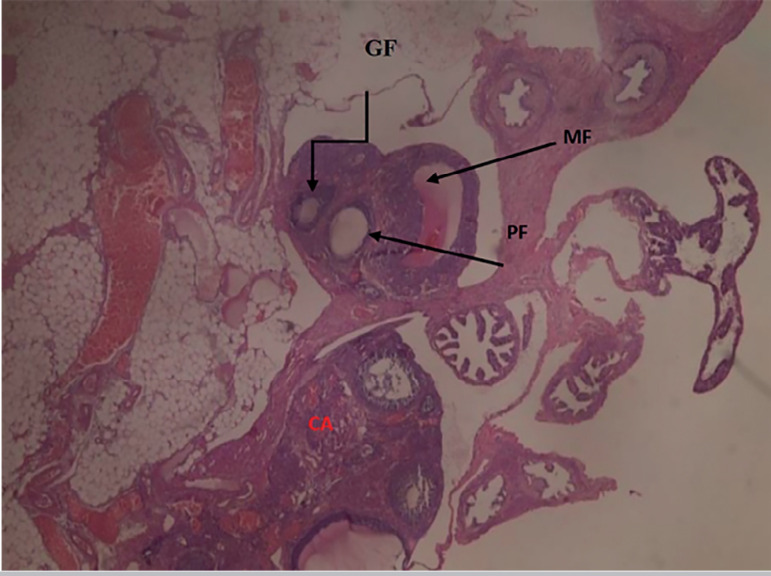
Photomicrograph section of a normal control showing ovarian tissue with primary follicle (PF), secondary follicle (SF) and mature follicles (MF) with antrum, with granulosa and theca cells. There is corpus albicans (CA) and blood vessels showing normal ovarian activity. H&E x100

**Figure 2:**
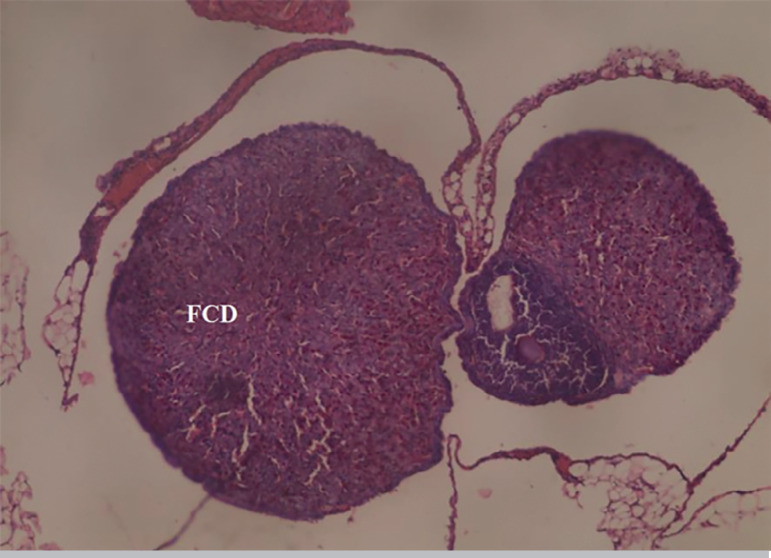
Photomicrograph section of ovary from Cadmium treated group showing severe tissue necrosis and follicular cell degeneration (FCD), atresia and no formation of new follicles. There is total degeneration of follicular cells with no significant ovarian activity. H&E x100

**Figure 3:**
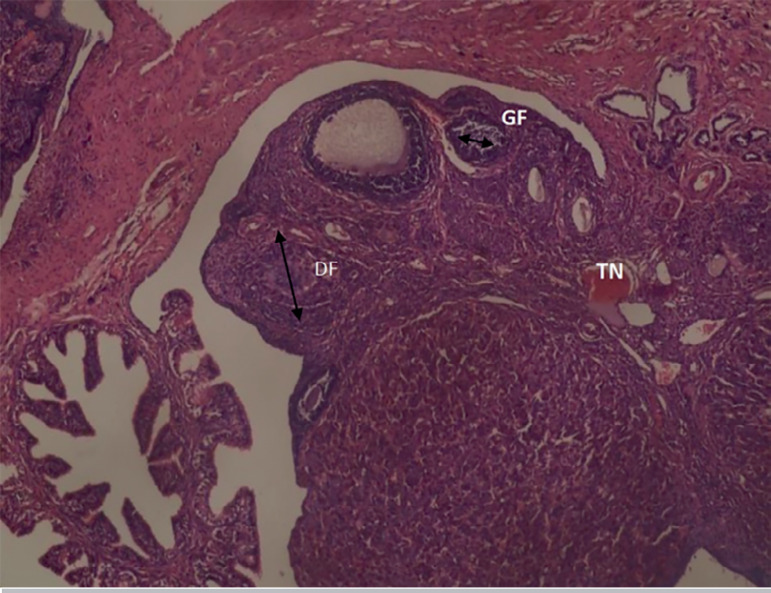
Photomicrograph section from he group treated with 2 mg/kg bw cadmium plus 50 mg/kg bw of X. aethiopica, showing few germinal follicles, primary follicle and numerous degenerated follicles with connective tissue necrosis (TN). H&E x100

**Figure 4:**
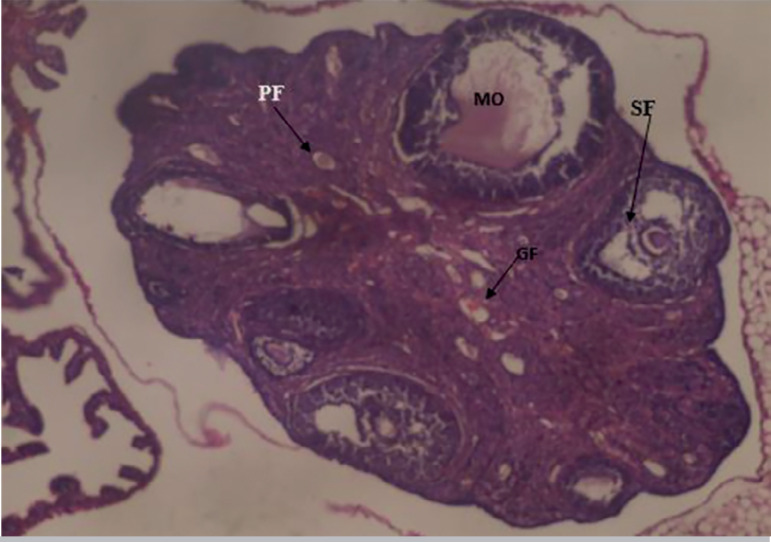
Photomicrograph section of ovarian tissue from the group treated with 100 mg/kg bw X. aethiopica seed extract showing tissue protection with primary follicles (PF), secondary follicles (SF) and mature oocytes (MO). There are new germinating follicular cells (GF). This demonstrates ovarian tissue protection against cadmium-induced cellular damage. H&E x100

**Figure 5:**
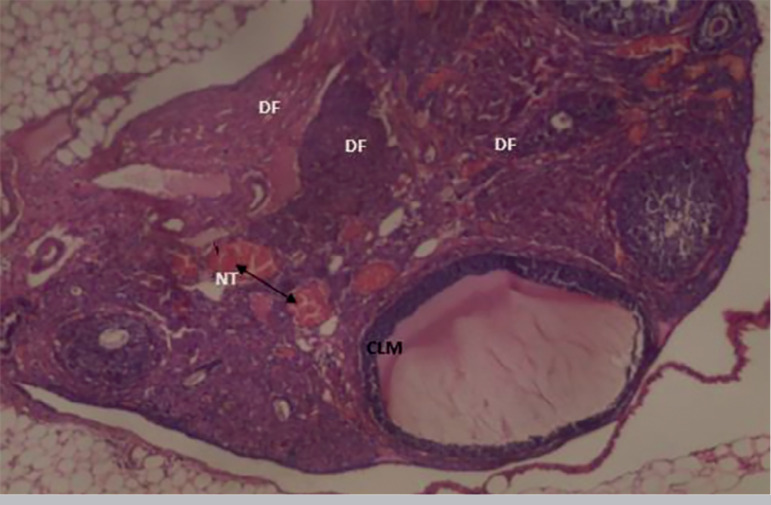
Photomicrograph section of ovarian tissue from 100 mg/kg bw X. aethiopica seeds extract-treated group only showing mature oocyte, primary and secondary follicles (DF) H&E x 100

The results obtained from the photomicrograph sections (Figure 1) showed the control group with ovarian tissue having clear primary follicle (PF), secondary follicle (SF) and mature follicles (MF) with normal antrum, granulosa and theca cells. There is also corpus albicans (CA) and blood vessels showing normal ovarian activity. Figure 2 shows a photomicrograph section of ovary from the cadmium-treated group, only showing severe tissue necrosis and follicular cell degeneration (FCD), as well as atresia and no formation of new follicles. There is total degeneration of follicular cells, with no significant ovarian activity. Images from treatments with *Xylopia aethiopica* extracts revealed an increasing number of primary and secondary follicles, as well as other cellular constituents. The increase was dose-dependent as higher doses of extracts presented with a larger number of regenerating cellular constituents (Figure 3, 4, 5).

## DISCUSSION

The findings showed a significant body weight reduction (*p*<0.05) with the cadmium chloride-treated group which became progressive with the duration of administration. This is in agreement with the findings of Osifo & Iyawe (2018). Varying doses of *Xylopia aethiopica* seeds extract showed a significant increase (*p*<0.05) in body weight. Studies by Singh *et al*. (2011) revealed that oxidative stress could be the cause of the weight reduction seen with cadmium chloride toxicity. Therefore, the weight reduction found in this study could be attributed to oxidative stress following cadmium chloride toxicity, affecting the brain function, nutritional uptake in the gastrointestinal system and metabolic activities. The significant weight gain seen in the *Xylopia aethiopica* seeds extract treated group could probably be the result of its ability to reduce the oxidative stress induced by cadmium chloride plus its high content in carbohydrates and phytochemicals like alkaloids, terpenoids, polyphenols, kauranes (a type of diterpenes, called xylopic acid and kaurenoic) (Omeh *et al.,* 2014).

The results obtained for the hormonal analysis showed a significant increase (*p*<0.05) in serum levels of FSH and LH in the groups treated with cadmium chloride, compared to the control group (Table 2). This could be the result of a negative feedback mechanism of reduced progesterone and estrogen levels from the ovarian tissues due to the detrimental impact of cadmium chloride toxicity. This is in agreement with findings from Dix-Cooper & Kosatsky (2018), who revealed that cadmium chloride alters steroidogenesis, induces pregnancy loss, causes disorders of the menstrual cycle and reproductive hormones, delayed puberty (Chedrese *et al*., 2006; Gallagher & Kovach, 2010). However, contrary to the work of Nampoothiri & Gupta (2006), who showed that cadmium chloride significant decreases gonadotrophin levels, thereby disrupting the activities of steroidogenic enzymes. Although this discrepancy may be the result of the study duration, the route of administration and the dose of cadmium chloride administered. The groups receiving seed extracts of *X. aethiopica* showed a significant reduction in gonadotrophins (Table 2). These finding is consistent with the study from Nnodim *et al*. (2013), who revealed that 80 mg/kg of *X. aethiopica* dried fruit extract caused a significant reduction in FSH levels in male Wistar rats, and also the study from Onuka *et al*. (2017), who reported that *X. aethiopica* extract caused a significant reduction in serum LH level during the proestrus and estrus phases, and attributed the action of the extract to the high content of saponins, having the ability to block the release of LH from gonadotropes. These findings may be a result of the *X. aethiopica* antioxidant ability to reduce the oxidative stress induced by cadmium chloride. Several studies have revealed the detrimental role of oxidative stress in causing hormonal imbalance and dysfunctions, or acted via the inhibition of the rate-limiting enzyme, HMG-CoA reductase, in the biosynthesis of cholesterol, which is the precursor molecule for all steroid hormones. HMG-CoA reductase catalyze the conversion of HMG-CoA to mevalonic acids (Omeh *et al.,* 2014).

Results from the histological analysis revealed that tissue protection with primary follicles, secondary follicles and mature oocytes as well as the formation of germinating follicular cells has been demonstrated in all the groups treated with *X. aethiopica*. This improvement is seen to be dose-dependent.

## CONCLUSION

This study has shown that *X. aethiopica* has ameliorative effect against cadmium chloride on ovary. These effects may be attributed to its antioxidants property. Further studies should be carried out to isolate the active components responsible for this antioxidant effect and to evaluate the likely mechanism of action.
